# Crystallography of quasiperiodic moiré patterns in homophase twisted bilayers

**DOI:** 10.1107/S2053273324012087

**Published:** 2025-01-30

**Authors:** Marianne Quiquandon, Denis Gratias

**Affiliations:** a CNRS UMR 8247 Institut de Recherche de Chimie ParisTech 11 rue Pierre et Marie Curie 75005Paris France; Universidad del País Vasco, Spain

**Keywords:** crystallography, bilayers, quasiperiodicity, 0-lattice

## Abstract

This paper discusses the geometric properties and symmetries of general moiré patterns generated by homophase bilayers twisted by rotation 2δ. These patterns are generically quasiperiodic of rank 4 and result from the interferences between two basic periodicities incommensurate to each other, defined by the sites in the layers that are kept invariant through the symmetry operations of the structure. These invariant sites are distributed on the nodes of a set of lattices called Φ-lattices – where Φ runs on the rotation operations of the symmetry group of the monolayers – which are the centers of rotation 2δ + Φ transforming a lattice node of the first layer into a node of the second. It is demonstrated that when a coincidence lattice exists, it is the intersection of all the Φ-lattices of the structure.

## Abstract 2

The quasiperiodic nature of moiré patterns in homophase twisted bilayers is analyzed to understand the underlying basic periodicities and symmetries of homophase twisted bilayers built on any 2D structure.

## 1. Introduction

Twisted homophase bilayers – made of the superimposition of two twisted 2D monolayers of the same phase – have been a very active research area since the discovery in 2010 of a localization of Dirac electrons in graphene bilayers (Trambly de Laissardière *et al.*, 2010 ▸, 2012 ▸), chalcogenide [see for instance Venkateswarlu *et al.* (2020 ▸)] and heterophase bilayers [see for instance Le Ster *et al.* (2019 ▸)], leading to strong electronic correlations and superconductivity (Lopes dos Santos *et al.*, 2007 ▸, 2012 ▸; Suarez Morell *et al.*, 2010 ▸; Bistritzer & MacDonald, 2011 ▸; Kim *et al.*, 2017 ▸; Yankowitz *et al.*, 2019 ▸). Although many theoretical and experimental studies have been carried out to better understand this new electronic localization, the question of what in the complex symmetry properties of twisted bilayer 2D materials is at the origin of this electronic localization is still partially unanswered. It is therefore essential to combine different approaches, both theoretical and experimental, to study together the crystallographic structures and the electronic properties.

With respect to the crystallographic point of view discussed here, the interesting twisted bilayers are those showing a small twist rotation of about 1° in graphene [see for instance Campanera *et al.* (2007 ▸), Kim *et al.* (2017 ▸), Cao *et al.* (2018 ▸), Tarnopolsky *et al.* (2019 ▸)] which generates very large moiré cells with respect to the monolayer unit cell and thus induces quantum interferences at the range of the moiré cell. Our goal is to build an exhaustive crystallographic description of twisted homophase bilayers whatever the atomic structures of their monolayers and for any twist rotation. We presented the first part of this work in a previous article (Gratias & Quiquandon, 2023 ▸), referred to as G&Q in the following, which discussed the crystallographic properties of the specific twisted bilayers that show a coincidence lattice. The present article is a direct continuation, constituting the second part of the work; it focuses on the geometric properties of the moiré patterns associated with *generic twisted homophase bilayers that have no coincidence lattices* or, from a practical point of view, no coincidence lattice of unit-cell size comparable with that of the monolayer.

Beyond the introduction and the conclusion, the paper is divided into three main parts.

The first part deals with the basic tools used to characterize the geometry of homophase bilayers. This is achieved by the knowledge of the half angle of rotation δ between the two layers and the rigid-body translation τ of the second layer with respect to the first.

The second part discusses the important concept of the geometrical locus of invariant points in bilayers, called here the *zero locus*, with the introduction of the Φ-lattices that are a generalization of the 0-lattice discovered by Bollmann (1967 ▸, 1970 ▸) long ago. Two of these sets of invariant sites define lattices that are the basis of the almost-periodicity of the moiré patterns generated by the interference between the two monolayers.

In the third and last part, specific attention is paid to connecting these Φ-lattices to the coincidence lattice, when it exists, which is shown to be a subgroup common to all those of the Φ-lattices and the initial lattices of the two monolayers. On the other hand, these Φ-lattices are distributed in space in a special way in the case of the three high-symmetry quasi­periodic cases: a square quasicrystal generated by two rectangular structures rotated by π/2, an octagonal quasicrystal generated by two square structures rotated by π/4 and a dodecagonal quasicrystal generated by two hexagonal structures rotated by π/6.

## 2. The basic tools

As discussed in G&Q, we make use of complex numbers which are the natural tool to use for describing 2D crystallography that requires manipulation of isometries and translations as defined in the *International Tables for Crystallography* (Hahn, 2005 ▸). It is remarkably efficient to establish explicit algebraic expressions of the crystallographic properties of twisted bilayers. Indeed, depending on the context, complex numbers 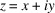

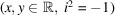
 are either a Euclidean vector space of dimension 2, 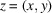, where the usual scalar product 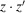 is the real part of the product 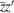 [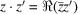], or a commutative algebra over the real numbers with operators corresponding to a 2 × 2 real matrix: 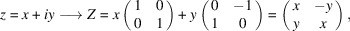
as exemplified by the rotation operator 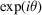: 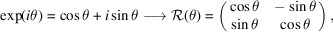
and the conjugation operator 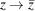 corresponds to the 2 × 2 real matrix
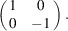
Lattices are defined by choosing parameter 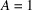 as the unit length along the real axis whatever the system and parameter *B* as the complex number 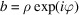, where ρ is its length in 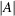 units and φ its angle with *A*: 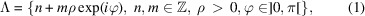
with unit-cell area 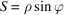.

The reciprocal lattice 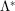 is easily found to be
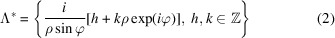
and the standard symmetry operations of 2D crystallography are defined as follows: (i) a translation 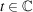 acts as 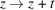; (ii) a rotation ϕ around the origin acts as 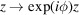; (iii) a mirror along the direction θ and passing through the origin acts as 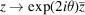.

### 2.1. Defining ideal homophase twisted bilayers

We define a homophase twisted bilayer as superposition in the (*x*, *y*) plane of two ideally thin atomic monolayers of the same structure of lattice Λ and 2D space group 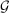 of point group Γ, twisted with respect to each other by a rotation α and displaced by a rigid-body translation **T**, noted 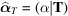 and acting as 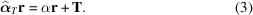
Of course, because of the intrinsic symmetry 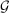 of the monolayer, the transformation from layer 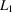 to 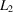 can equivalently be characterized by any operator of the set 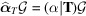 that we define as the *transformation set* (see G&Q and references therein). The inverse transformation set characterizing the transition from 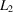 to 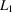 is defined by 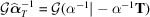. We designate by 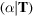 the rotation–translation operator of the transformation set 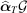 that has the *smallest absolute value of the rotation angle* α. We will use throughout the paper the half rotation angle 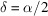 instead of α which is actually the pertinent rotation parameter, as will be made clear next.

**Figure 1:**
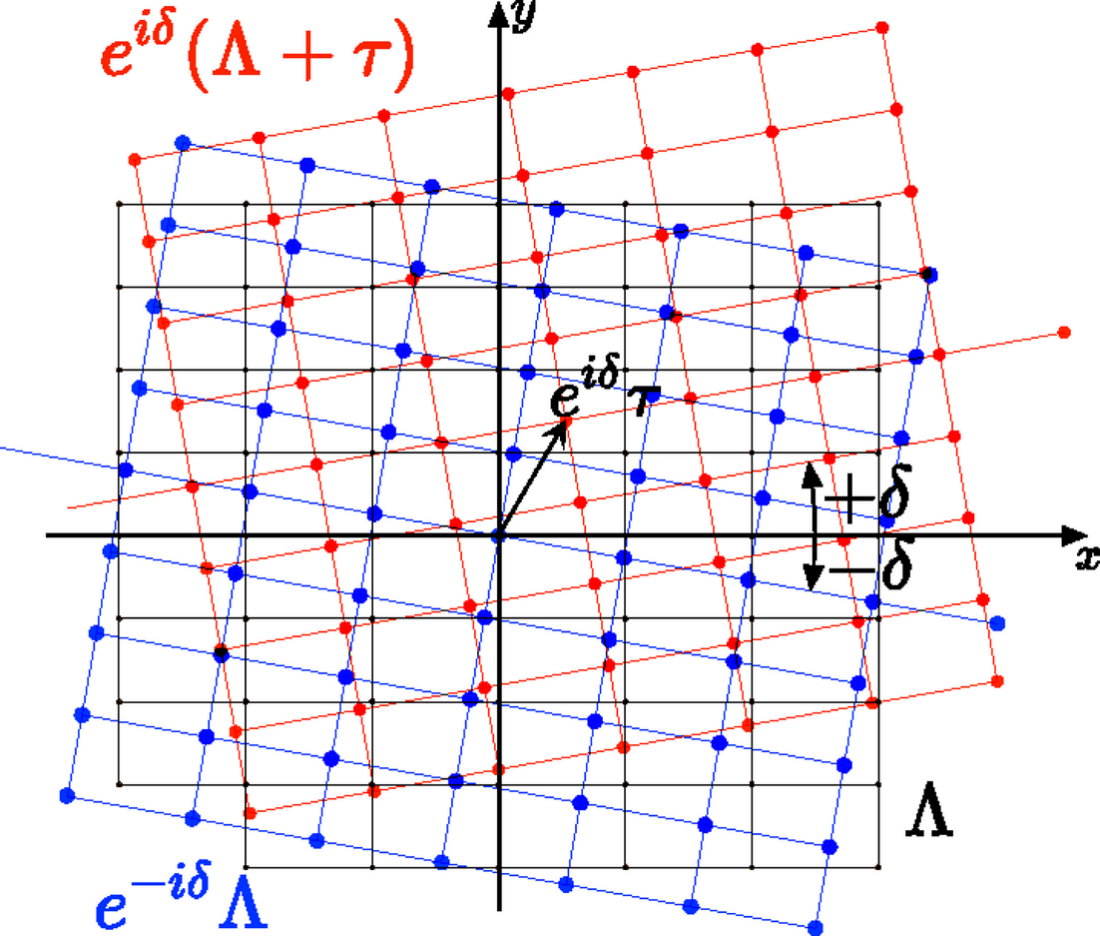
The reference frame (*x*, *y*) is defined by the initial monolayer structure of lattice 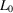 (black). The first layer 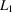 (blue) is a copy of 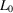 rotated by 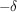 around the origin, 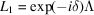. The second layer 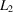 (red) is a copy of 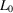 displaced by a rigid-body translation 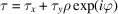 and then rotated by 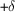, leading to 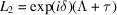.

The homophase twisted bilayer is constructed as follows. We make a first copy of the original monolayer structure 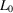 of lattice 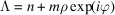, in black on Fig. 1 ▸, which we rotate by 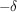 around the origin, leading to 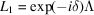 in blue on Fig. 1 ▸. The second layer 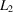 in red on the figure is a copy of 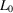 first displaced by the rigid-body translation 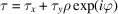 and then rotated by 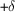 [this choice of first translating 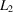 before the rotation is arbitrary; it was chosen for simplicity since then τ is directly read in 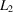 coordinates with a total operation that factorizes into 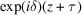], so that finally 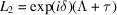. With these notations, the complete transformation (3) 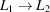 can be written as
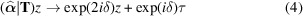
in the reference frame of the first layer 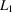.

For obvious symmetry reasons, and as illustrated on Fig. 1 ▸, we choose the reference frame (*x*, *y*) (in black on the figure) along the bisector axes of the two layers. Any position *z* in the original monolayer 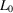 transforms into the two homologous points 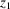 and 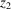 of, respectively, 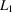 and 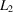 according to
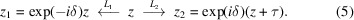


### 2.2. Generating moiré patterns

To generate Young–Fresnel interference patterns, we associate to each monolayer a continuous function 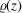 defined by the Fourier sum: 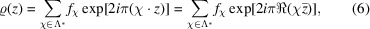
where the vectors χ run on the nodes of the reciprocal lattice 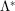 and 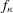 are * ad hoc* Fourier coefficients reflecting its intrinsic symmetry. [In practice, only a few Fourier terms are enough for illustrating a moiré effect, as soon as *all* the reciprocal vectors of a given orbit are taken into account in order to ensure the characteristic function 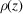 properly reflects the symmetries of the space group of the structure.] For ease of reading, we use here the standard notation of the scalar products 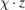 in the Fourier arguments rather the explicit form 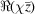.

The superposition of these characteristic functions 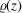 of each layer generates more or less complicated interference effects between the two functions, which we will refer to as *moiré patterns* whatever the values of the rotation angles, not only the small ones [see for instance Miller *et al.* (2010 ▸)].

The simplest interference function 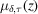 at point *z* is obtained by adding the value of 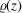 at point 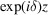 [inverse of 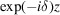] for 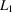 to that at point 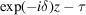 [inverse of 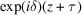] for 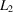: 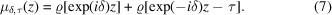
The function 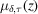 is visualized throughout this paper by its modulus 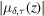 using the standard gray scale where black corresponds to zero and white to the largest value.

### 2.3. Moiré patterns at small rotations

**Figure 2:**
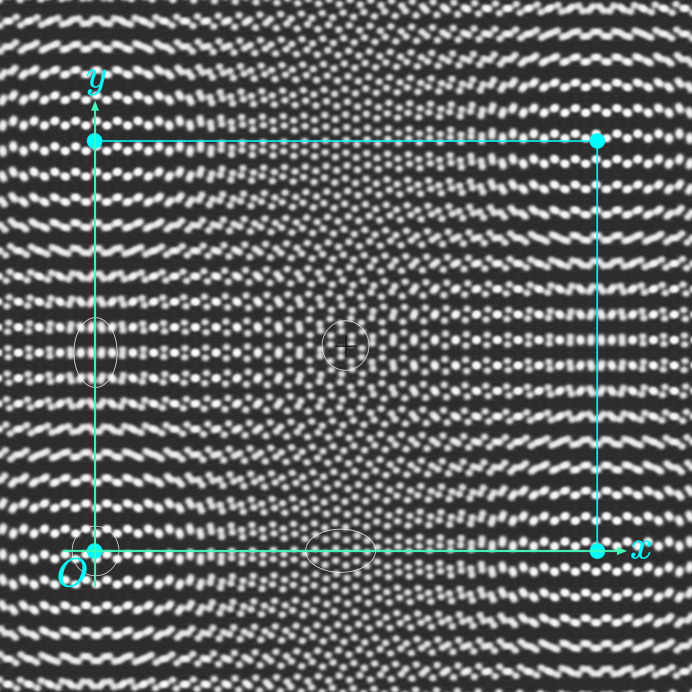
Bilayer of a rectangle structure of group *pmg* (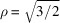) rotated by δ = 1.45°. At 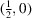 of the unit cell of the pseudo-periodic lattice 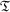, in cyan, the two structures are displaced by 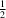 of Λ in the *y* direction and vice versa. The three main bilayer structures obtained by simple translation between the two *pmg* layers are clearly identified: at (0, 0) the initial structure *pmg*, at 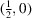 another structure *pmg*, at 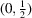 a structure *pmm* and at 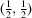 a structure *pgm*.

We first recall that, for a small rotation 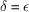, the interference function can be approximated as
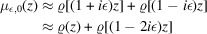
and thus
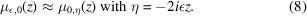
This is the usual result that the interference function 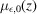, for a 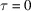 rigid-body translation, is well approximated to first order in ε by the function 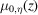, where η *varies explicitly with z* according to 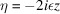. At a given point *z*, located with respect to the axis of rotation, the moiré pattern obtained by a rotation ε is locally identical, at first order in ε, to the simple superimposition of the initial structure onto itself shifted by a translation depending on the point *z* under consideration, perpendicular to it and proportional to its distance to the rotation center, as illustrated in Fig. 2 ▸. Because of the translational symmetry of the structure, this displacement is bounded by the unit-cell vectors of the lattice Λ and cycles to zero periodically when 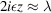, leading thus to an apparent periodicity 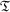 for the moiré pattern given by
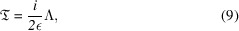
which is a copy of the initial lattice enlarged by 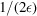 and rotated by 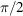 as exemplified in Fig. 2 ▸. This relation, as will be shown later, is indeed the approximate expression of the 0-lattice for small rotation angles.

Because of the presence of ε in the denominator, the periods of 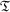 in relation (9) are large with respect to those of Λ and generally incommensurate with them. Although of little pertinence for such large differences in scale, a commensurability between these two lattices exists when 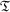 is a subgroup of Λ, * i.e.* when the coordinates of the unit cell of 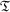 take integer values when expressed in the Λ unit cell [*A* = 1, *B* = 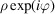]. From relation (9), this arithmetical condition translates as
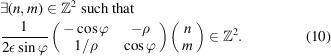
For Λ being an oblique lattice, there are no generic solutions for coincidence. For the rectangle system (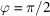), condition (10) leads to 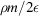 and 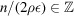. This imposes 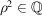, as already discussed in G&Q, and ε of the form 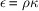 with 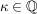. For the square system (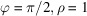), ε must simply be a rational number 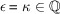. Finally, for the hexagonal system (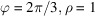), ε must be of the form 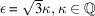. In all other situations, 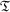 and Λ are incommensurate to each other and the periodicity of 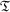 is not exact at the level of the atomic unit cell Λ. It is only an approximate periodicity of the bilayer that is truly quasiperiodic. This point will be discussed in Section 3.3 (Φ-lattices).

Examining the moiré pattern of bilayers with very small rotations is the most efficient way of revealing at once all possible bilayer structures generated by two identical layers translated from each other by a running offset [see for instance Kobayashi (1996 ▸)].

### 2.4. Symmetry of moiré patterns

The specific case where the bilayer has a coincidence lattice – and thus a standard space group – is exhaustively treated in G&Q and is not discussed here.

Since a generic moiré pattern is quasiperiodic of rank 4, we reduce the symmetry operations of interest to those point symmetries of the intensities of the Fourier spectrum of the bilayer in the spirit of Bienenstock & Ewald (1962 ▸), who demonstrated long ago that reciprocal space is where symmetry is best described and understood. These symmetry transformations leave invariant the correlation functions to any finite order and are based on the notion of *indistinguishability*, as discussed by Mermin (1992 ▸), rather than of superposition. As clearly explained by Lifshitz (2011 ▸), this indistinguishability property is for continuous functions what the so-called property of *local isomorphism* (see Levine & Steinhardt, 1986 ▸; Socolar & Steinhardt, 1986 ▸; Lubensky *et al.*, 1985 ▸) is for the set of vertices of the tiling description, meaning that *any finite part of one set is to be found in the other with the same frequency and vice versa*.

Let *G* be the point group of the constitutive monolayer. The point group Γ of the bilayer with rotation 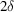 is given by the union of the intersection group 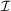 and the exchange set 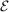
[see for instance Gratias *et al.* (1979 ▸), Gratias & Portier (1982 ▸), Gratias & Quiquandon (2020 ▸)] expressed in complex notations: 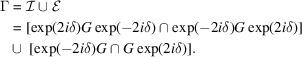
Since the rotation operations 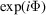 of *G* commute with 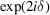, whatever the value of δ, all rotation operations of *G* belong to 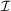 and therefore to the point group Γ of the bilayer. Concerning the mirrors in the exchange set 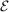, since 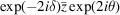 = 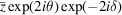, whatever the values of δ and θ, all mirrors in *G* are in the exchange set 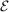 and thus belong to Γ so that eventually 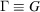; *the homophase bilayer has at least the same point symmetry as the constitutive monolayers, whatever the twist rotation*. This implies the variation domain of δ can be limited by the irreducible elementary domain of the Wigner–Seitz cell of the monolayer structure: 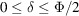, where Φ is the smallest rotation in *G*. Hence, δ being chosen positive, 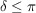 for the oblique system, 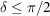 for the rectangle system, 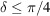 for the square system and 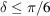 for the hexagonal system.

An additional symmetry arises in examining the superimposition of the diffraction patterns of two identical bilayers of a 2D structure with point group Γ and rotated with respect to each other by θ. The superimposition of the two moirés generates additional symmetries if the exchange set between the two moirés 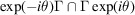 is not empty, *i.e.* if two rotations Φ and 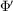 of Γ exist such that 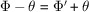 or when 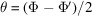 is half a rotation of the point group Γ of the monolayer struture. Let 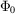 be the rotation of the smallest non-zero value in Γ. A special rotation symmetry of 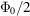 appears between two moiré patterns if they are generated, respectively, by rotations 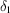 and 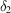 such that 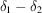 = 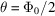, in particular when 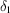 = 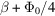 and 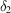 = 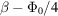 or equivalently 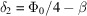 by exchanging 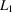 and 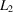 with 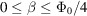.

**Figure 3:**
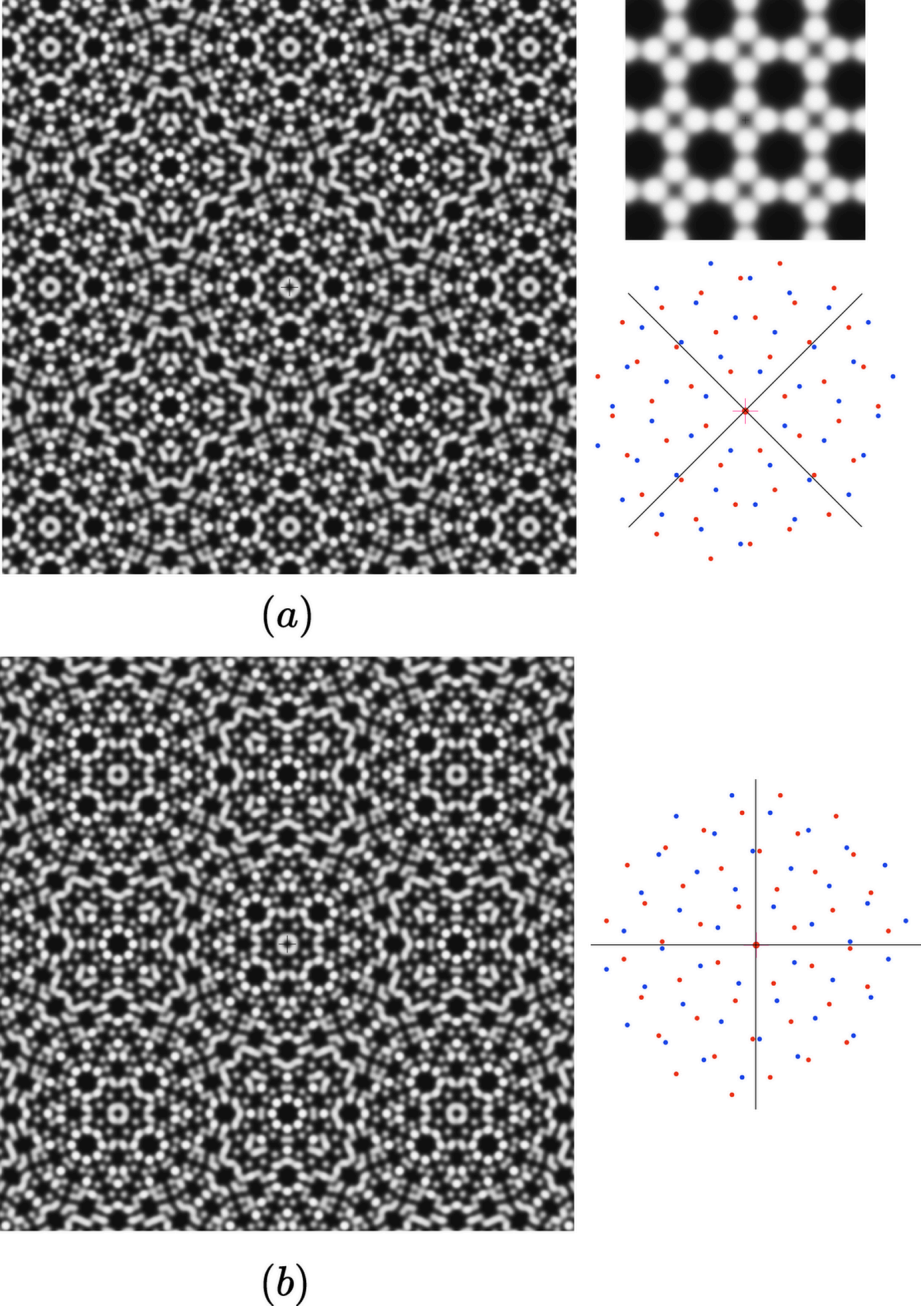
Example of a bilayer of a *p*4*mm* truncated square structure (inset on top right) with half rotation 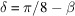 in (*a*) and 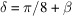 in (*b*) where β = 2° and τ = (0, 0). Although the global twist rotations differ by 8° between (*a*) and (*b*), the moiré patterns are identical and rotated by 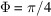 from each other, as shown on images and reciprocal lattices on the right.

This demonstrates that the moiré patterns 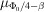 and 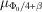 are locally isomorphic up to a global rotation of 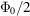 (and an exchange between 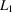 and 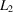 which is an intrinsic invariant of the moiré patterns). This is illustrated in Fig. 3 ▸ with a bilayer of a truncated square structure of group *p*4*mm* with 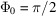, 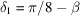 and 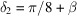 with β = 2°. Although the two bilayers differ here by only 8° in their twist angles, their corresponding moiré patterns are related to each other by a rotation of 45° whatever the value of β.

At the limit 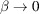, for 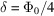, the two patterns merge into a unique one that acquires the additional symmetry rotation of 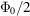 in its exchange set. This generates special high-symmetry quasicrystals such as a square quasicrystal from the superimposition by δ = π/4 of two identical rectangle structures, an octagonal quasicrystal from the superimposition of two square structures by δ = π/8 and a dodecagonal quasicrystal issued from two hexagonal structures rotated by π/12.

In summary, we note that, whatever the crystallographic system, the definition domain of δ actually reduces to 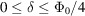 where 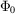 is the rotation of the smallest angular value (of highest order) in the group *G* of the monolayer. The δ values larger than 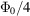, say 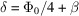, 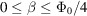, lead to moiré patterns that are locally isomorphic to those generated by 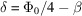, but rotated by 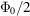. This leads to quite narrow boundaries for these irreducible values of δ:

For the point groups 1, *m*: 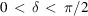.

For the point groups 2, 2*mm*: 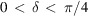.

For the point groups 4, 4*mm*: 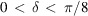.

For the point groups 3, 31*m*, 3*m*1: 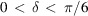.

For the point groups 6, 6*mm*: 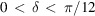.

The special high symmetries of the moiré patterns for twist rotations at the upper limit of the elementary domains are shown in Table 1 ▸. The high-symmetry patterns of the oblique and trigonal classes are trivially periodic with the same period Λ as the monolayer since the additional rotations belong to the invariance group of the lattice itself. In all other cases, the patterns are quasiperiodic with square, octagonal and dodecagonal point symmetries for, respectively, the rectangle, square and hexagonal systems. These cases are exemplified later in Section 4.2.

## 3. General moiré patterns

**Figure 4:**
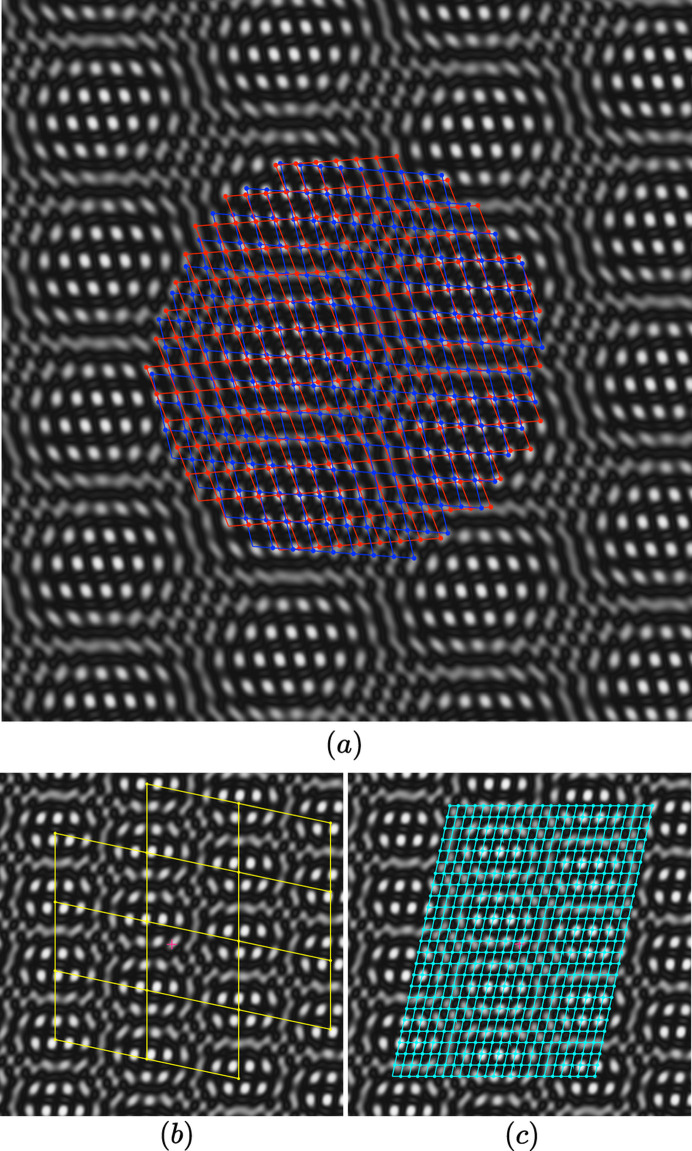
(*a*) Typical moiré pattern 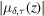 of a homophase bilayer made of an oblique structure *p*2 [ρ = 1.365, φ = 108°, δ = 4°, τ = (0.2, 0.3)_Λ_] with one atom per unit cell located on the twofold axis; the two lattices 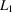 and 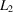 are drawn in, respectively, blue and red. (*b*) The yellow lattice corresponds to the half period 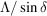 of the distribution of the moiré cells whereas in (*c*) the lattice in cyan of half period 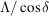 gives the internal structure of each cell.

For small twist rotations of a few degrees, as shown in Fig. 4 ▸ with δ = 4° for a bilayer made of an oblique structure of group *p*2 with one atom per unit cell, we typically observe a set of similar supercells containing details of the initial structure that are shifted by one half of the initial monolayer’s period from one cell to its neighbors. A careful examination of Fig. 4 ▸ shows that, although very similar, these supercells are not identical: the interference function is indeed an almost-periodic function of rank 4 in the general case and it is only when the twist rotation generates a coincidence lattice that the pattern reduces to a truly 2D-periodic function.

### 3.1. The interference function generating the moiré pattern

To quantify the almost-periodicity in the general case, we expand 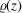 in its Fourier terms (6) in the calculation (4 ▸) leading to
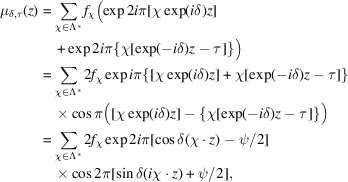
where 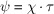 is the phase change induced by the rigid-body translation τ.

This result is the 2D version of the standard 1D interference phenomenon of two functions 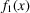 and 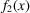 of close periodicities: 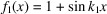, 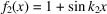, leading to 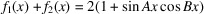
with 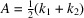 and 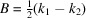.

Here, the interference function 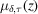 is the Fourier summation running on the reciprocal-lattice nodes 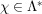 of terms that are products of two components:

(i) The term 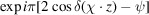 oscillating with half the periods of 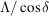.

(ii) The term 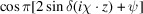 oscillating with half the periods of 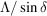 rotated by π/2 (because of the imaginary symbol *i* in the scalar product).

When δ is small, as shown in Fig. 4 ▸, the first term oscillates with periods of the same order of magnitude as the elementary unit cell because 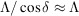, whereas the slowly oscillating term of periods 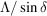 rotated by 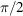 generates the long-distance interference effect responsible for the moiré phenomenon.

Understanding what specific geometric properties these two modulation periods in 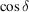 and 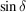 correspond to, in the general case of large rotations, requires a more detailed analysis of the geometry of the bilayer system, as discussed next.

### 3.2. Geometric locus of invariant points: the zero locus

The rotation from the first layer to the second leaves specific points of the plane invariant (staying at the same location during the transformation from one layer to the other), forming a geometric set of points we designate as the *zero locus* [see for instance Gratias *et al.* (1979 ▸)].

**Figure 5:**
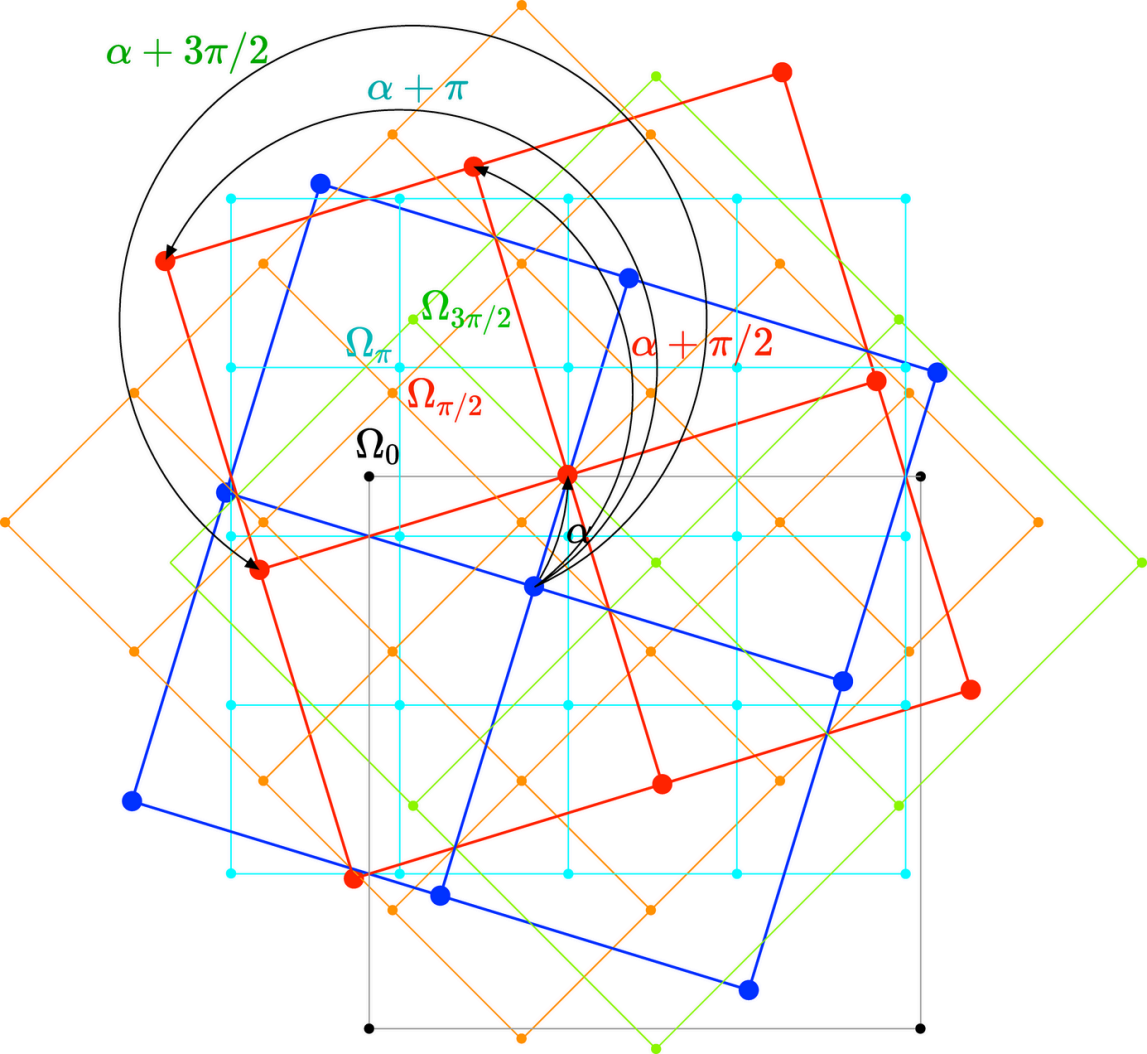
In a square structure, the blue layer transforms into the red one according to four different equivalent rotations: 1, a rotation α = 2δ around the center 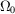, node of the 0-lattice (black); 2, a rotation 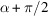 around the center 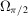, node of the 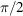 lattice (orange); 3, a rotation α + π around the center 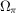, node of the π-lattice (cyan); 4, a rotation α + 3π/2 around the center 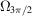, node of the 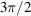-lattice (green). All these four lattices are part of the zero locus.

Because the layers are periodic, those invariant points repeat regularly all over the plane and are distributed on the nodes of lattices, as the so-called 0-lattice discovered and first discussed by Bollmann (1967 ▸, 1970 ▸) in his studies of the geometry of grain boundaries in metals. But this original 0-lattice is only a fraction of the whole zero locus: as already mentioned, the twist operation is characterized by an infinite number of equivalent operations that form the transformation set 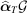: invariant points 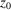 are those that transform into at least one of their equivalents: 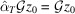, as exemplified on Fig. 5 ▸.

The zero locus is therefore the geometric locus of the carriers of all the reducible operations of the set 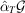, here a set of points (carriers of rotation axes) and straight lines (carriers of pure mirrors).

Let *z* be a generic point of the initial 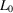 layer and 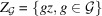 its orbit under the space group 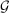.

The image of *z* in 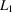, say 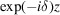, is a point 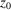 of the zero locus if it superimposes onto at least one, say 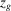, of its equivalents in 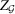 of 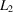: 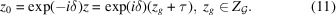
The elements 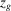 of the orbit 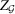 take two different forms according to the nature, rotation or mirror, of the implied symmetry operation *g* of 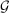:

(i) *g* is a rotation of angle Φ associated to a non-primitive translation *t* and a lattice translation λ: 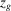 = 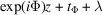;

(ii) *g* is a mirror oriented in the θ direction associated to a non-primitive translation *t* and a lattice translation λ: 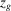 = 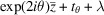.

The zero locus is the collection of the invariant points obtained by exploring all the points of 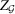 in equation (11).

### 3.3. The invariance by rotation: the Φ-lattices

**Figure 6:**
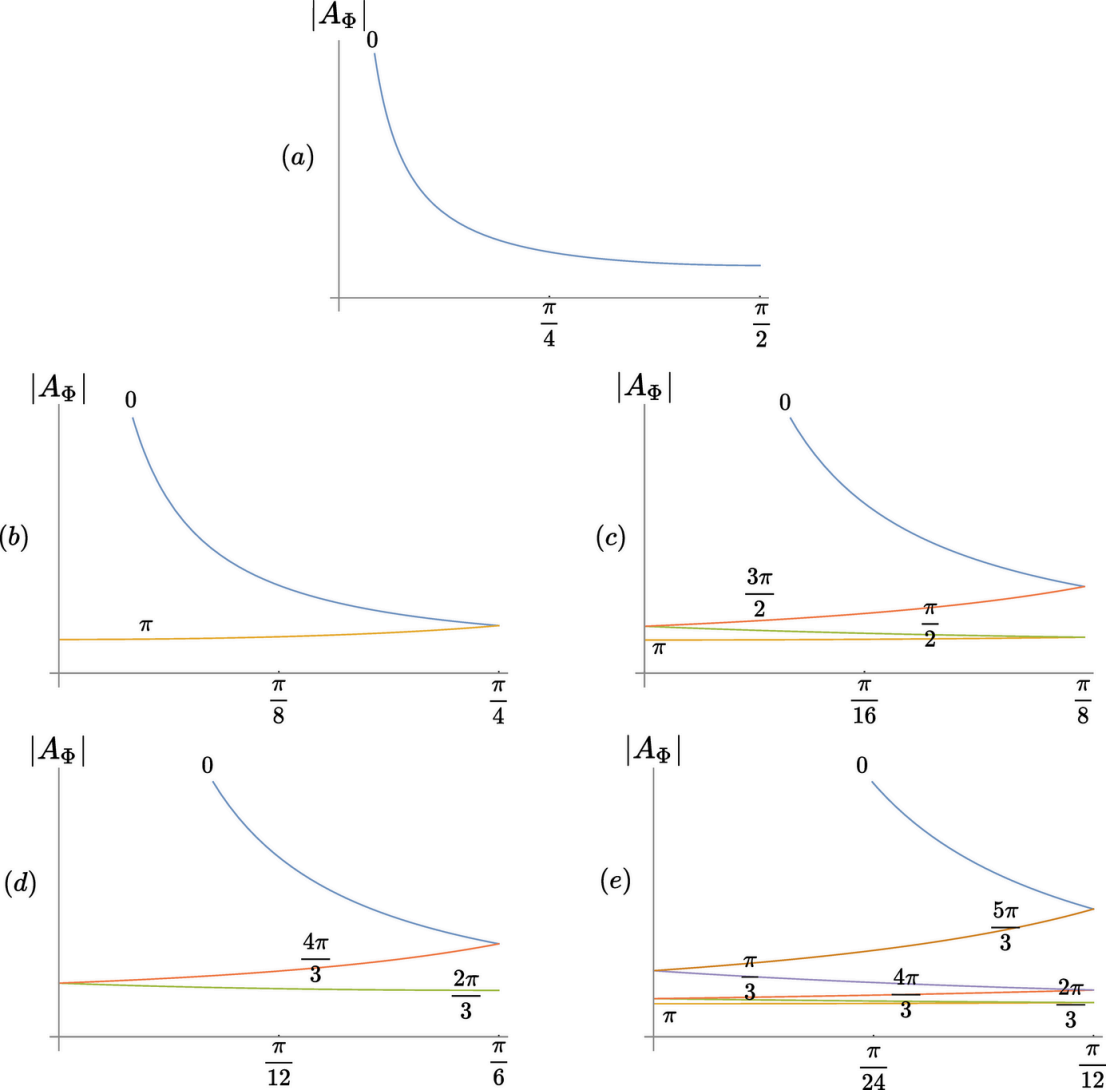
Variations of 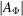 [relation (13)] versus δ according to the point symmetry of the crystalline system of the monolayer (regardless of whether the bilayer is periodic or not): (*a*) 1, *m*, (*b*) 2, 2*m*, (*c*) 4, 4*m*, (*d*) 3, 3*m*1, 31*m* and (*e*) 6, 6*m*. In all cases but the oblique and trigonal, the largest value of 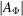 is obtained for 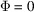 and the smallest for 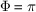.

Applying relation (11) in the case of 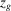 = 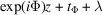, resulting from a rotation of angle Φ associated to a non-primitive translation 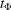 and a lattice translation λ, gives
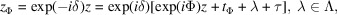
leading to
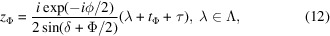
where 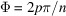 with *n* = 1, 2, 3, 4, 6 and 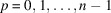: the set 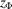 is a set of *sites specifically located in space* distributed on the nodes of a lattice isomorphic to Λ translated by 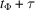 rotated by an angle of 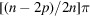 and scaled by 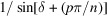. We will refer to this set as the Φ-lattice. We designate by
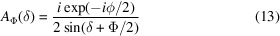
the (complex) prefactor term defining the Φ-lattices (see Fig. 6 ▸).

The simplest case of relation (12) is given by 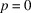, whatever the value of *n*, *i.e.* the identity operator up to a lattice translation, 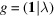 with 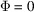 and 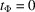, which is valid for any structure and corresponds to reducing the orbit of *z* to its lattice translation part only. This generates the geometric set
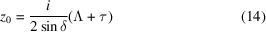
which is the analytical expression of Bollman’s 0-lattice in 2D and is to be compared with expression (9) for small δ: this is a lattice, an image of Λ displaced by τ, rotated by π/2 and scaled by 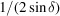. Each of its nodes is an equivalent axis of rotation 2δ that superimposes the first layer on top of the second one. Expression (14) reduces to that obtained by Aragón *et al.* (2019 ▸), 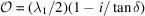 with 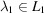 for 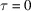 and expressed in the 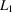 reference frame. It corresponds also to the standard formula of moiré patterns which gives the spacing *D* between two successive maxima in the moiré pattern generated by two 1D lattices of period *p* rotated by 2δ: 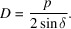


The second simplest case of relation (12), which applies to all lattices but only to centrosymmetric structures, is given by *p* = 1, 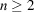, *i.e.*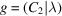, with 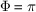, with the twofold axis 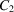 located at the origin 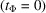, in which case we find
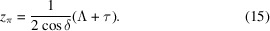
For *n* = 4, *i.e.* in the square system, two additional lattices (*p* = 1, 3) are obtained for the rotation operators 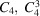, where 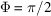 and 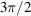 with the fourfold axis located on the origin, 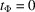.

For *n* = 3, *i.e.* for the trigonal point symmetry in the hexagonal system, we find the 0-lattice (*p* = 0) plus the two lattices *p* = 1, 2 associated to 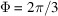 and 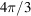 and for *n* = 6, *i.e.* the hexagonal point symmetry, the addition of the π-lattice (*p* = 3) and two new ones (*p* = 1, 5) associated to 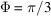 and 5π/3. In all these cases, the symmetry axes can be chosen to be located at the origin 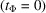.

The Φ-lattices are the natural generalization of Bollman’s 0-lattice which corresponds to 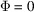. Each node of a Φ-lattice is a center of rotation of angle 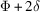 that relates 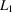 to 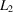, as exemplified in Fig. 5 ▸ and discussed in Appendix *A*[App appa]. The various Φ-lattices implied in the zero locus as a function of the space group of the layers are given in Table 2 ▸.

It is useful to make the following remarks that hold whatever the value of the twist angle 2δ:

(i) The Φ-lattices exist whatever the value of the rotation δ and vary smoothly with δ; they must be considered together as a fraction of the invariant carriers of the set 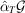.

(ii) They are the locations where the characteristic function 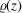 of one layer takes the same value with the same phase in the other layer; they are sets of *well defined fixed positions* distributed on the nodes of lattices depending on the point symmetry of the layers.

(iii) Two points connected by a Φ-lattice translation are generally *not* crystallographically equivalent with respect to the symmetries of the layers or the bilayer, in particular the existence or not of a coincidence lattice.

(iv) When a coincidence lattice exists, it is the common sublattice of all Φ-lattices and 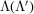 (see Section 4.1 for an example).

### 3.4. Visualizing the zero locus

**Figure 7:**
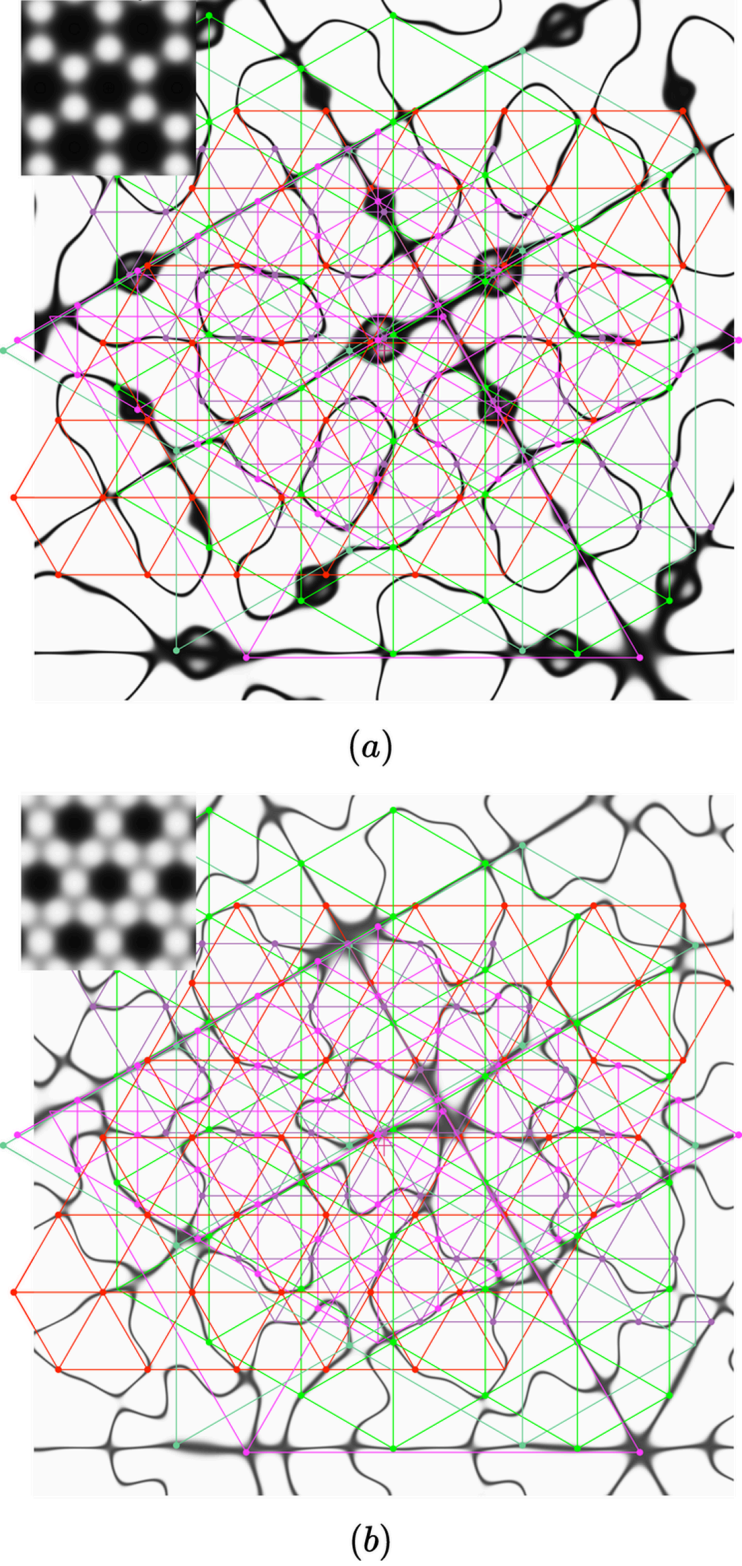
Details of the functions 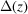 of honeycomb (*a*) and kagome (*b*) bilayers for δ = 10° and 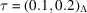 show how the zeros follow curves (in black) in the plane that are quite different. However, since the six Φ-lattices (in colors) of the hexagonal system are common to both structures, their nodes (colored dots) are distributed equally well on the zeros of the two functions: they are all at the intersections of the two black curves (*a*) and (*b*).

To visualize the zero locus as a whole, we use the real positive function
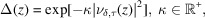
where 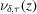 = 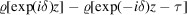 is the difference between the two characteristic functions of the monolayers. This function 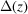 takes its maximum value 1 when 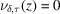 and decreases rapidly to 0 everywhere else depending on the value of κ [in practice, 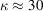 for normalized functions 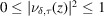]. This leads to density maps with well defined curves drawn in color or in black for 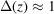 on a white background everywhere else when 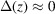. They reveal the regions of the bilayer plane where the function 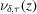 is close to zero, and give a quite faithful view of the complete zero locus of the invariant points in the twist rotation of the bilayer, as illustrated in Fig. 7 ▸. We note that, whatever the values of δ and τ and the atomic structure of the layer, as long as it has the Φ rotational symmetry, the difference function 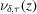 cancels out on the nodes of the corresponding Φ-lattice.

### 3.5. Identifying the basic almost-periods of the moiré patterns

We note from the examination of Fig. 4 ▸ that, for small δ, the two basic almost-periods of the moiré pattern are those of the 0- and the π-lattices:

(i) The fast oscillating term with periods 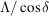 of the same order of magnitude as the elementary unit cell is twice the period of the π-lattice.

(ii) The slowly oscillating term of periods 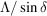 rotated by π/2 responsible for the long-distance interference moiré effect of supercells is twice the period of the 0-lattice.

These two sets characterize entirely the moiré effect generated by the superimposition of two identical twisted layers; the 0-lattice is a geometrical invariant for all structures whereas the π-lattice is also an invariant for 2, 2*m*, 4, 4*m* and 6, 6*m* but only with respect to lattices for 1, *m*, 3 and 3*m* point symmetries.

For large δ values, the moiré effect of supercells diminishes and transforms into more intricate patterns as the two basic periods become closer to each other.

### 3.6. The mirror invariants

We find from relation (11) that mirror-invariant points 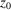, if they exist, are such that
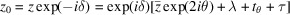
or
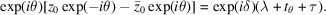
Such invariant loci are straight lines along the direction θ passing through fixed points, say 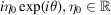: 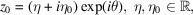
provided that at least one lattice translation 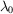 is such that
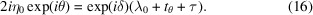
This relation (16) has a simple geometric interpretation: the right-hand side represents a node 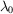 of 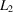 displaced by 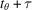 expressed in the 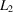 unit-cell coordinate system; the left-hand side describes a straight line Δ along the direction 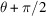 for 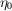 running in 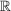.

In practice, we consider the lattice of 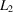 and draw the line Δ passing through the point of coordinates 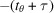. The solutions of relation (16), if any, are the lattice nodes 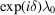 of 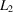 that are located on Δ: 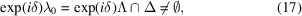
in which case 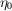 is half the distance between two consecutive such nodes.

**Figure 8:**
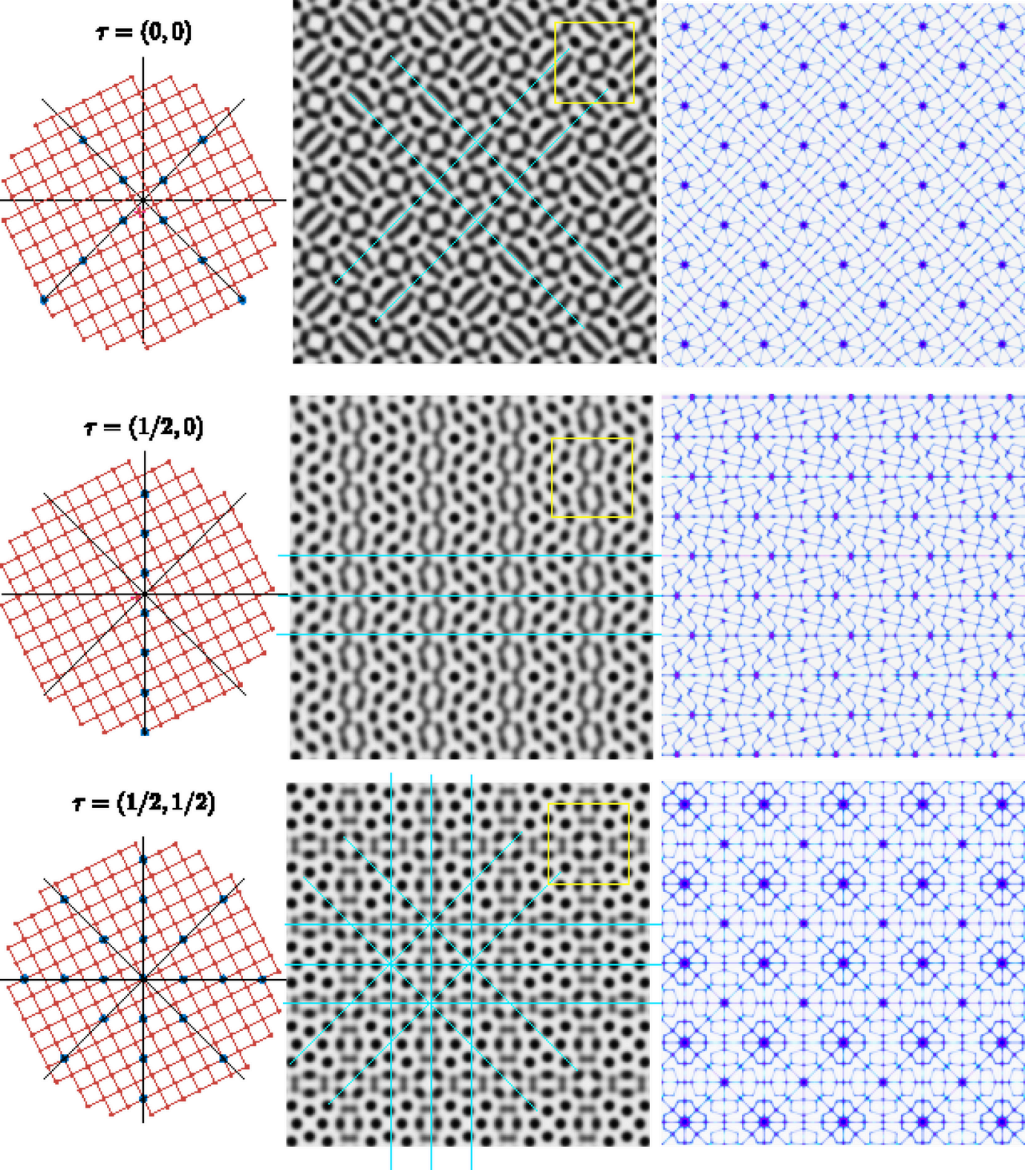
Bilayer of structure *p*4*gm* for the rotation δ = 18.435° with coincidence lattice 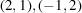 of the unit cell in yellow on the moiré patterns. On the left, the straight lines Δ in black are the traces of the mirrors *m* and 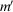; the lattice of 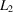 is displaced by the non-primitive translation *t* = (1/2, 1/2) associated to the mirrors in *p*4*gm*. For τ = (0, 0), the Δ lines hit lattice nodes along the diagonal directions generating pure mirrors along these directions for a bilayer symmetry *p*4*gm*. For τ = (1/2, 0), they hit lattice nodes only in the *y* direction which generates a set of mirrors along the *x* direction for a bilayer symmetry 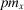. For τ = (1/2, 1/2) they hit lattice nodes on all mirror directions of the square for a bilayer symmetry *p*4*mm*. In blue, on the right column, the zero locus.

This is exemplified in Fig. 8 ▸ with a bilayer made of two identical layers of space groups *p*4*gm*. Choosing the origin on a fourfold axis leads to a non-primitive translation 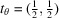 for all mirrors of the unit cell. We therefore draw the lattice of 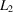 displaced by *t* and check condition (17) with respect to the value of τ. For τ = 0, intersection points are found along both diagonal directions, generating pure mirrors along these directions.

Applied to point symmetry only where 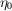, *t* and τ vanish, relation (16) reduces to
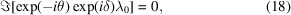
leading to
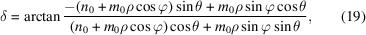
with 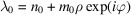, 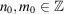 and coprimes.

This is relation (3) of G&Q which defines the rotation angles δ that generate coincidence lattice rows in any 2D crystallographic system but the oblique one.

We observe that when 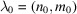 is a solution of (18), then all the nodes of the row 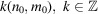, are also solutions. Hence, by choosing 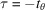, we obtain an infinite number of parallel invariant straight lines associated to the mirrors consistently with the existence of a coincidence row. For the square and hexagonal systems, satisfying relation (18) is enough to generate 2D coincidence lattices, as illustrated in Fig. 8 ▸. In this case, the part of the zero locus issuing from mirrors is the traces of the reducible mirrors of the bilayer space group that depends on the value of τ (see Section 3.2 in G&Q).

In the general case of non-coincidence, and because of (17), the mirrors are of secondary importance in the determination of the zero locus. Since any generic straight line intersects a discrete set of lattice nodes on at most one point, there are only a few exact straight lines in the zero locus. However, there are infinitely many lattice nodes of 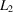 that are close to a straight line within a thickness ε.

## 4. Φ-Lattices, coincidence lattices and high-symmetry quasiperiodic patterns

The set of the Φ-lattices presents specific interesting geometric properties in two cases: when the bilayer has a coincidence lattice; when the bilayer presents an extra symmetry generating a high-symmetry quasiperiodic moiré pattern.

### 4.1. Coincidence lattices

As already discussed in G&Q, there are no generic possible 2D coincidence lattices for the monolayer structures belonging to the oblique systems. For the rectangular system 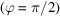 of unit cell 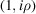, 2D coincidence lattices exist only if ρ is the square root of a rational number: 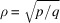 with 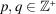.

When the rotation δ generates a bilayer with a coincidence lattice, the Φ-lattices take special forms according to the crystalline system of the monolayer of unit cell 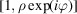. Since we are searching for lattice properties (translation group–subgroup relations), we can ignore the rigid-body translation and choose τ = 0.

The coincidence lattice 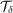 is the set of lattice translations that are common to both 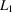 and 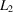, 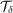 = 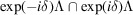. Applying the alignment property (see Appendix *A*[App appa]) to that pair of superimposed nodes with zero length implies that all corresponding nodes of the ϕ-lattices must converge at the coincidence point itself so that this coincidence site belongs to each of the Φ-lattices: the coincidence lattice 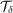 is a common subgroup of all the Φ-lattices. Such a point 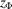 that belongs simultaneously to each of the Φ-lattices would be the center of rotation of two nodes 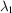 and 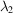 connected by several *different* rotation values of Φ. This implies these two nodes 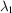 and 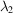 are superimposed at this same point 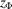 and therefore 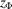 belongs to the coincidence lattice. Thus, we obtain the general group relation:
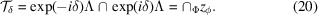
To quantify this general relation (20) for each crystal system, we recall that, in all but the oblique system, the rotation δ defined by tan δ = *m*ρ sin φ/(*n* + *m*ρ cos φ) superimposes the lattice node (*n*, *m*) onto (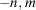).

We define σ = 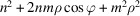.

For the rectangle system, coincidence lattices 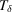 exist for 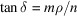 with unit cells 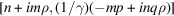, where γ = gcd(*mp*, *nq*). The Φ rotations of the rectangular system are 0 (monolayer structure with point group *m*) and π (monolayer structure with point group 2*mm*), leading to the two Φ-lattices:
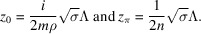


It is easily shown that 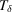 is the subset of 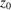 with coordinates 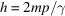, 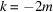 and is the subset of 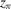 with coordinates 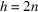, 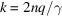. The two integers *n* and *m* being coprimes, the coincidence lattice 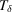 is thus the largest common sublattice of both 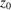 and 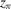 in addition to the lattices of 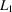 and 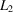.

For the square system (ρ = 1, φ = π/2), coincidence lattices take the simple form 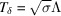 with tan δ = *m*/*n*. In addition to 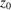 and 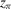, there are two new Φ-lattices associated to the rotations 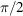 and 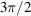 of the square system: 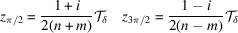
and therefore
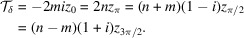


In the hexagonal system 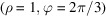, coincidence lattices 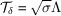 arise for 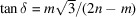.

For monolayer structures of trigonal point groups 3, 31*m* and 3*m*1, we have
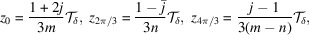
and backwards
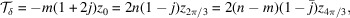
and for monolayer structures with hexagonal point groups 6 and 6*mm*, the addition of
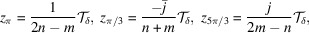
and thus
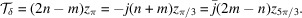


### 4.2. High-symmetry moiré patterns

**Figure 9:**
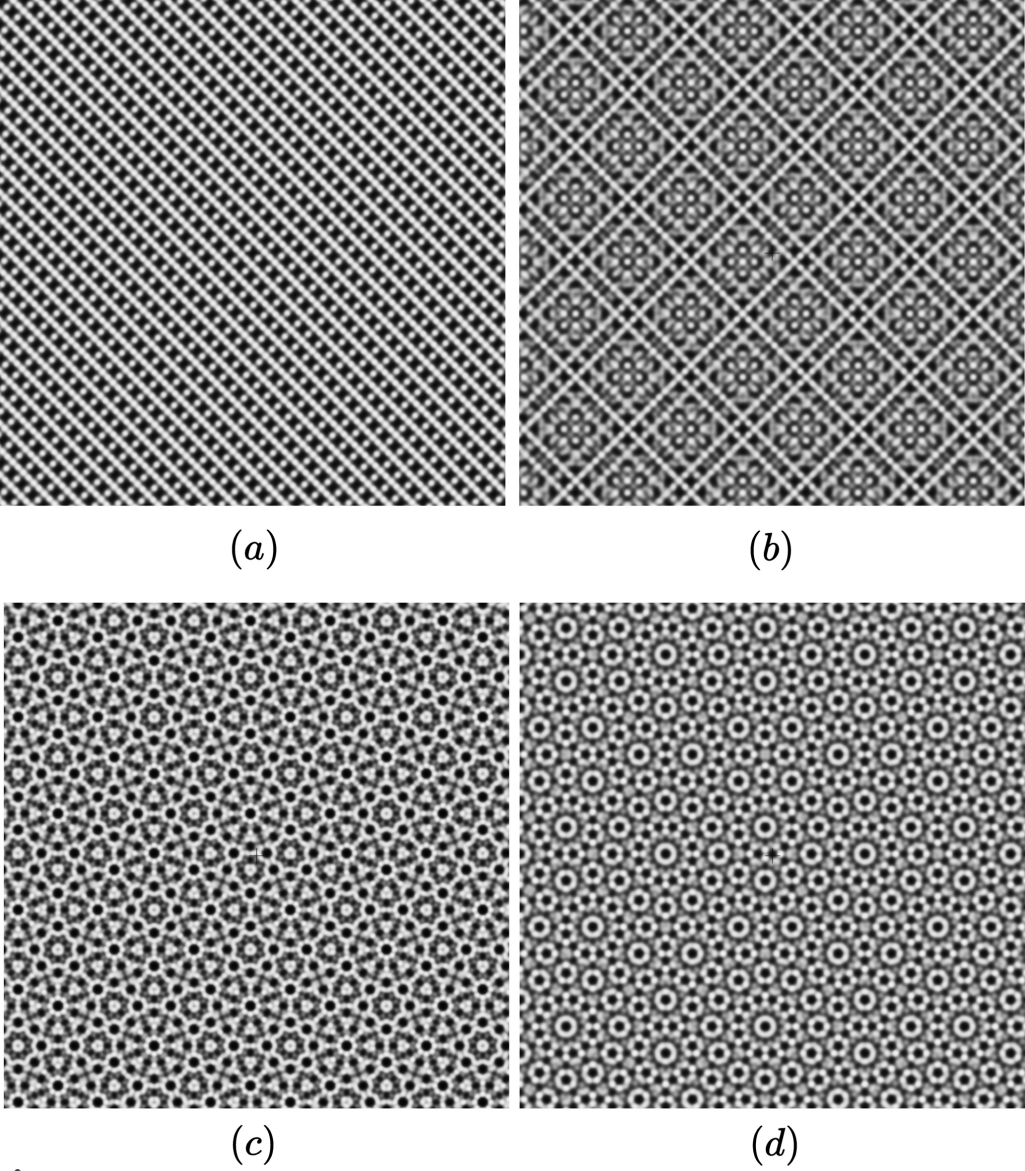
(*a*) Monolayer structure *p*2*mm*, ρ = 3/2; (*b*) bilayer of structure (*a*) with δ = π/4: a quasiperiodic square pattern; (*c*) bilayer of structure truncated square *p*4*mm* δ = π/8: a quasiperiodic octagonal pattern; (*d*) bilayer of structure honeycomb *p*6*mm* δ = π/12: a quasiperiodic dodecagonal pattern.

As shown in Table 1 ▸ and already discussed in Section 2.4, non-trivial high-symmetry quasiperiodic patterns are generated for specific twist angles in the rectangle, square and hexagonal systems as shown in Fig. 9 ▸. The complex prefactor 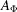 defined by relation (13) designates equivalently the unit vector 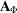 of the corresponding Φ-lattice.

**Figure 10:**
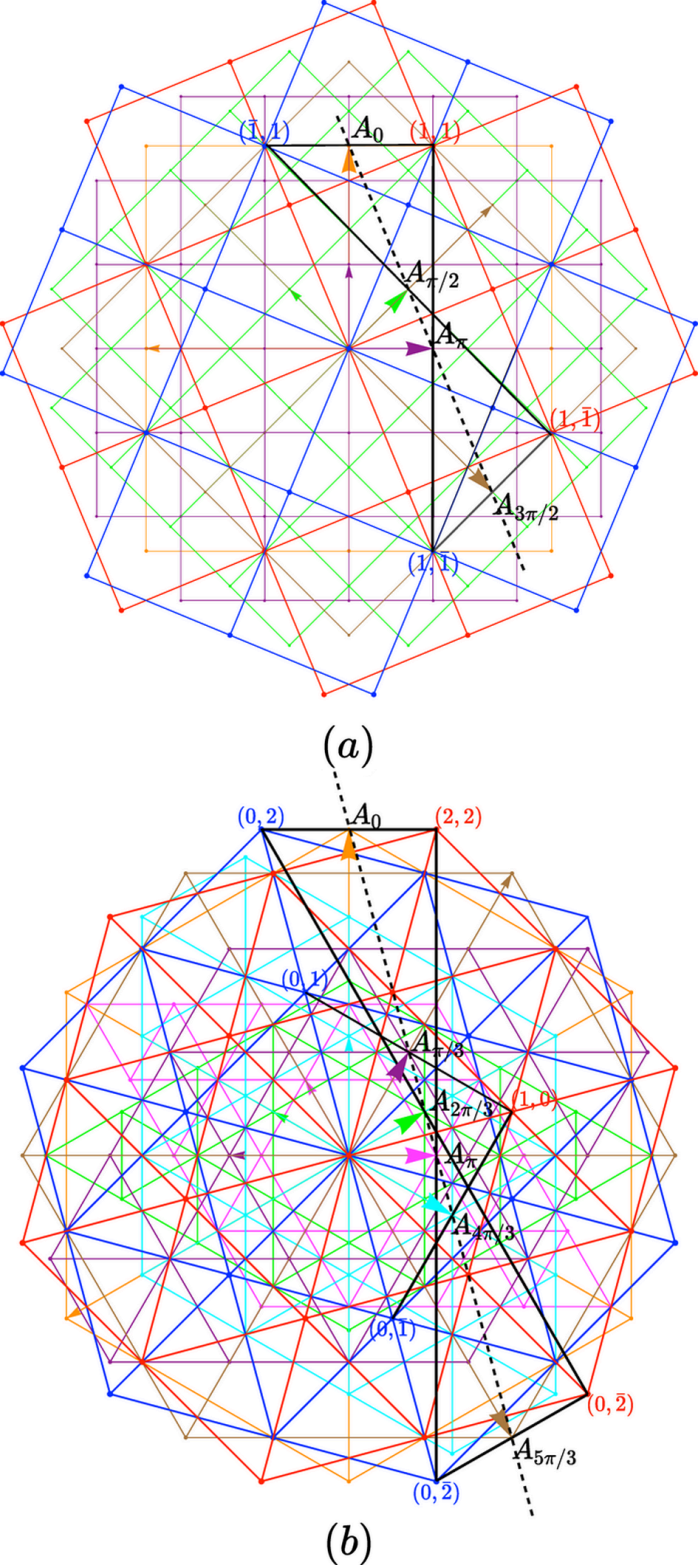
The complete set of Φ-lattices for the octagonal (*a*) and dodecagonal (*b*) quasiperiodic patterns (see Fig. 11 ▸ for a general discussion). In these high-symmetry cases, the prefactors 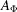 and thus the **a** unit-cell vectors of the corresponding Φ-lattice (with 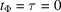) are all half an integer sum of a node of 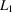 with a node of 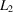.

We observe that, for these specific high-symmetry cases, *the Φ-lattices are all half projections of nodes of the 4D lattice*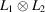 as shown on Fig. 10 ▸ for the square and hexagonal systems: 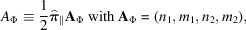
where 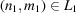 and 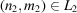.

#### 4.2.1. The rectangle system

The rectangle system with 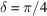 generates a square quasiperiodic pattern with two identical Φ-lattices rotated by π/2: 
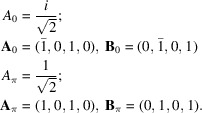


#### 4.2.2. The square system

The square system with δ = π/8 generates octagonal quasiperiodic patterns with four Φ-lattices of identical size two by two and rotated by π/4: 
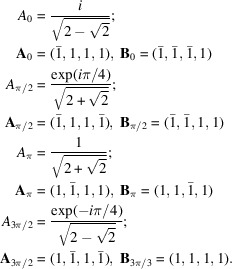
We notice that the ratio of the lengths of the lattice parameters is
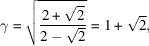
which is the basic inflation factor of the Ammann–Beenker octagonal tiling.

#### 4.2.3. The hexagonal system

The hexagonal system with δ = π/12 generates dodecagonal quasiperiodic patterns with six Φ-lattices of identical size two by two and rotated by π/6: 
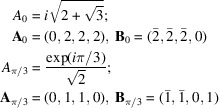

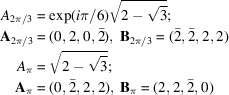

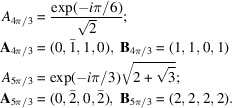
The ratios between the lengths of the lattice parameters are
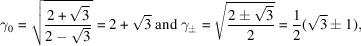
where here too 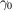 is the standard inflation factor of the dodecagonal tiling.

## 5. Conclusion

General moiré patterns of twisted homophase bilayers are quasiperiodic functions of rank 4 that can be described as the superimposition of two basic 2D-periodic functions: the first with a short period 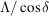 and the second with a long period 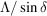 rotated by π/2, corresponding to the periodically spaced sites, called here Φ-lattices, of invariant points during the twist rotation of, respectively, the shortest and the largest periodicities. Every node of a Φ-lattice is a center of rotation 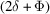 that transforms a node of the lattice of the first layer into a node of the lattice of the second layer and the coincidence lattice is the largest common subgroup of the Φ-lattices as expressed in relation (20).

The point symmetry of general moiré patterns is defined as the set of isometries of the global superimposition of the diffraction intensity spectra of the two layers; it is identical to the point group of the monolayer except for the holohedral structures (2*mm*, 4*mm*, 6*mm*) at the upper limits of the twist angle that generate an additional rotation of twice the order of the initial monolayer. In all cases, these point symmetries are to be understood relative to quasiperiodicity and correspond to the invariance of the correlation functions of any finite order of whatever property of the monolayer; they do not imply exact superimposition of the moiré pattern onto itself. Further work is in progress to examine how to connect moiré patterns of general bilayers with the usual tiling description developed for simple 4D quasicrystals.

## Figures and Tables

**Figure 11:**
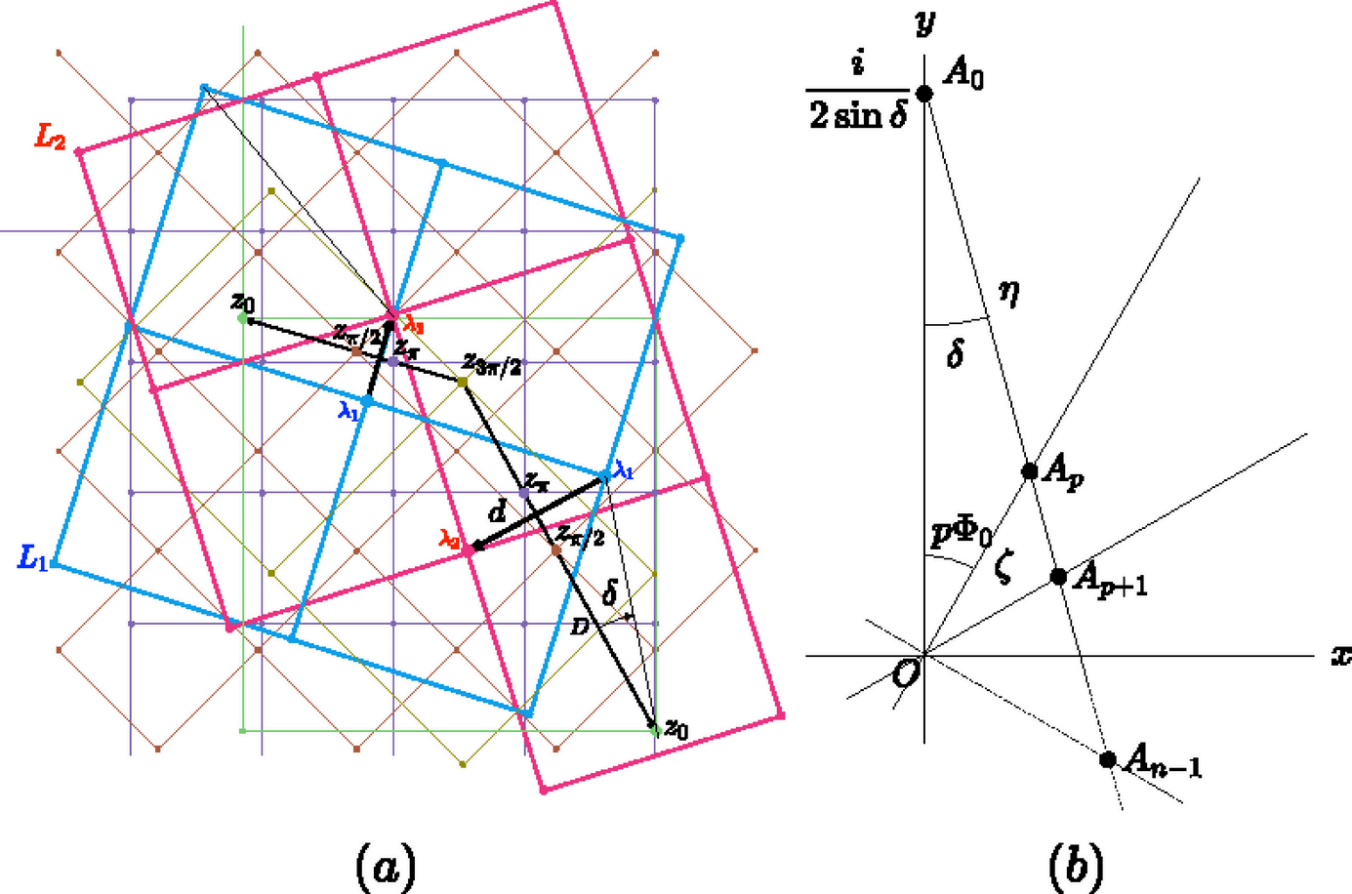
(*a*) Any lattice node of 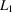 (blue) is transformed into any lattice node of 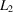 (red) by a rotation of 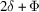 around a node of the corresponding Φ-lattice; therefore these rotation centers, each being a node of a given Φ-lattice, are aligned along the perpendicular bisector of the considered pair, as shown here for the square system. (*b*) The prefactors of the Φ-lattices associated to a given rotation order *n* align along a straight line of angle δ with the vertical axis. This property is directly observed on the drawing of the Φ-lattices for 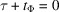 where the prefactors 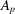 are then the locations of the nodes (1, 0) of each Φ-lattice, here for the square system with δ = 17.56° (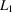 in blue and 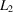 in red) with the four Φ-lattices: 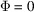 (green), π/2 (orange), π (purple) and 3π/2 (brown).

**Table 1:** Special high symmetry of bilayers with specific twist rotation 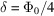 The general patterns are quasiperiodic except for the cases *n* = 1 and *n* = 3 where the additional symmetry belongs to the point symmetry of the lattice of the monolayer (noted with a star).

*n*	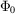	Space group 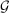	δ	Point symmetry
1	2π	*p*1	π/2	2 (oblique*)
1	2π	*pm*, *pg*, *cm*	π/2	2 (rectangle*)
2	π	*p*2, *p*2*mm*, *p*2*mg*, *p*2*gg*, *c*2*mm*	π/4	4 (square)
3	2π/3	*p*3, *p*31*m*, *p*3*m*1	π/6	6 (hexagonal*)
4	π/2	*p*4, *p*4*mm*, *p*4*gm*	π/8	8 (octagonal)
6	π/3	*p*6, *p*6*mm*	π/12	12 (dodecagonal)

**Table 2:** Φ-Lattices as a function of the space group 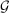 of the constitutive monolayer of homophase bilayers

Space group 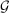	Φ-Lattice(s)	Part of Fig. 6 ▸
*p*1, *pm*, *pg*, *cm*	0	(*a*)
*p*2, *p*2*mm*, *p*2*mg*, *p*2*gg*, *c*2*mm*	0, π	(*b*)
*p*4, *p*4*mm*, *p*4*gm*	0, π/2, π, 3π/2	(*c*)
*p*3, *p*31*m*, *p*3*m*1	0, 2π/3, 4π/3	(*d*)
*p*6, *p*6*mm*	0, π/3, 2π/3, π, 4π/3, 5π/3	(*e*)

## Appendix A Some geometric properties of the Φ-lattices

The very basic property of the Φ-lattices is that any two nodes  and  of, respectively,  and  are deduced from each other by a rotation of 2δ + Φ around one specific site  of the corresponding Φ-lattice:

leading to

Thus, owing to the fact that :

which corresponds as required to formula (12) with .

As an immediate consequence, a new alignment property states that the  sites all align on the perpendicular bisector of the segment  whatever the values of δ and τ. Let  be the length of the segment , then each of the centers of rotation  is located on the perpendicular bisector of the two nodes at a distance , as shown in Fig. 11 ▸(*a*). In particular, when , *i.e.* when  coincides with , all these Φ-lattice sites converge to the coincidence point.

Another property derived from the geometric alignment between Φ-lattice nodes is the following. We note that the term  defined by relation (13) can be read in vector form as the  unit-cell vector of this lattice. We claim that for a given *n* and δ, the points  corresponding to  for  with  align along a straight line passing through  with the angle δ to the *y* axis as shown on Fig. 11 ▸(*b*). This results from the previous Φ-lattice node alignment property applied for the case where τ = 0 and the two nodes  and  are, respectively, (0, 0) of  and (1, 0) of . This is easily analytically demonstrated by considering Φ as a variable, say 2*t*, and rewriting equation (13) as a definition of the parametrized curve in 2D:

the computation of the derivative of which leads to

which is a constant with respect to *t*. The parametrized curve is therefore a straight line of slope  passing through the point  for *t* = 0.

Assuming backwards the point  to be on this line, we thus have  together with the elementary geometric equalities from Fig. 11 ▸(*b*):

leading to η = ; therefore, ζ =  and thus

which is expression (13), as expected. This remarkable linearity is illustrated in Fig. 11 ▸(*b*) in the case of the square system for a generic twist rotation δ = 17.56°.

## References

[bb1] Aragón, J. L., Naumis, G. G. & Gómez-Rodríguez, A. (2019). *Crystals*, **9**, 519–531.

[bb2] Bienenstock, A. & Ewald, P. P. (1962). *Acta Cryst.* **15**, 1253–1261.

[bb3] Bistritzer, R. & MacDonald, A. H. (2011). *Proc. Natl Acad. Sci. USA*, **108**, 12233–12237. 10.1073/pnas.1108174108 PMC3145708 21730173

[bb4] Bollmann, W. (1967). *Philos. Mag.* **16**, 363–381.

[bb5] Bollmann, W. (1970). *Crystal Defects and Crystalline Interfaces*. Springer-Verlag.

[bb6] Campanera, J. M., Savini, G., Suarez-Martinez, I. & Heggie, M. I. (2007). *Phys. Rev. B*, **75**, 235449–235462.

[bb7] Cao, Y., Fatemi, V., Fang, S., Watanabe, S., Taniguchi, T., Kaxiras, E. & Jarillo-Herrero, P. (2018). *Nature*, **556**, 43–50. 10.1038/nature26160 29512651

[bb8] Gratias, D. & Portier, R. (1982). *J. Phys. Colloq.* **C6**, C6-15–C6-24.

[bb9] Gratias, D., Portier, R., Fayard, M. & Guymont, M. (1979). *Acta Cryst.* A**35**, 885–894.

[bb10] Gratias, D. & Quiquandon, M. (2020). *Crystals MDPI*, **10**, 560–574.

[bb11] Gratias, D. & Quiquandon, M. (2023). *Acta Cryst.* A**79**, 301–317. 10.1107/S2053273323003662 PMC10317138 37265049

[bb12] Hahn, T. (2005). Editor. *International Tables for Crystallography*, Vol. *A*, *Space-Group Symmetry*, 5th ed. Heidelberg: Springer.

[bb13] Kim, K., DaSilva, A., Huang, S., Fallahazad, B., Larentis, S., Taniguchi, T., Watanabe, K., LeRoy, B. J., MacDonald, A. H. & Tutuc, E. (2017). *Proc. Natl Acad. Sci. USA*, **114**, 3364–3369. 10.1073/pnas.1620140114 PMC5380064 28292902

[bb14] Kobayashi, K. (1996). *Phys. Rev. B*, **53**, 11091–11099. 10.1103/physrevb.53.11091 9982681

[bb15] Le Ster, M., Maerkl, T., Kowalczyk, P. J. & Brown, S. A. (2019). *Phys. Rev. B*, **99**, 075422.

[bb16] Levine, D. & Steinhardt, P. J. (1986). *Phys. Rev. B*, **34**, 596–616. 10.1103/physrevb.34.596 9939667

[bb17] Lifshitz, R. (2011). *Isr. J. Chem.* **51**, 1156–1167.

[bb18] Lopes dos Santos, J. M. B., Peres, N. M. R. & Castro Neto, A. H. (2007). *Phys. Rev. Lett.* **99**, 256802. 10.1103/PhysRevLett.99.256802 18233543

[bb19] Lopes dos Santos, J. M. B., Peres, N. M. R. & Castro Neto, A. H. (2012). *Phys. Rev. B*, **86**, 155449. 10.1103/PhysRevLett.99.256802 18233543

[bb20] Lubensky, T. C., Ramaswamy, S. & Toner, J. (1985). *Phys. Rev. B*, **32**, 7444–7452. 10.1103/physrevb.32.7444 9936890

[bb21] Mermin, D. (1992). *Phys. Rev. Lett.* **68**, 1172–1175. 10.1103/PhysRevLett.68.1172 10046098

[bb22] Miller, D. L., Kubista, K. D., Rutter, G. M., Ruan, M., de Heer, W. A., First, P. N. & Stroscio, J. A. (2010). *Phys. Rev. B*, **81**, 125427–125433.

[bb23] Socolar, J. E. S. & Steinhardt, P. J. (1986). *Phys. Rev. B*, **34**, 617–647. 10.1103/physrevb.34.617 9939668

[bb24] Suárez Morell, E., Correa, J. D., Vargas, P., Pacheco, M. & Barticevic, Z. (2010). *Phys. Rev. B*, **82**, 121407–121411.

[bb25] Tarnopolsky, G., Kruchkov, A. J. & Vishwanath, A. (2019). *Phys. Rev. Lett.* **122**, 106405. 10.1103/PhysRevLett.122.106405 30932657

[bb26] Trambly de Laissardière, G., Mayou, D. & Magaud, L. (2010). *Nano Lett.* **10**, 804–808. 10.1021/nl902948m 20121163

[bb27] Trambly de Laissardière, G., Mayou, D. & Magaud, L. (2012). *Phys. Rev. B*, **86**, 125413–125420.

[bb28] Venkateswarlu, S., Honecker, A. & Trambly de Laissardière, G. (2020). *Phys. Rev. B*, **102**, 081103.

[bb29] Yankowitz, M., Chen, S., Polshyn, H., Zhang, Y., Watanabe, K., Taniguchi, T., Graf, D., Young, A. F. & Dean, C. R. (2019). *Science*, **363**, 1059–1064. 10.1126/science.aav1910 30679385

